# Plasma Extracellular Vesicles Biomarkers Linked to Lower Muscle Mass, Function and Physical Performance in Sarcopenia

**DOI:** 10.1002/jcsm.13784

**Published:** 2025-03-31

**Authors:** Ji Yeon Kim, Tae‐Hwan Gil, Hyo Gyeong Lee, Ji‐Won Shin, Dong‐Hyun Jang, Hyeon Soo Kim, Seung Shin Park, Sang Wan Kim, Chan Soo Shin, Sung Hye Kong, Ok Hee Jeon

**Affiliations:** ^1^ Department of Biomedical Sciences Korea University College of Medicine Seoul Republic of Korea; ^2^ Department of Anatomy Korea University College of Medicine Seoul Republic of Korea; ^3^ Department of Internal Medicine Seoul National University Hospital Seoul Republic of Korea; ^4^ Department of Internal Medicine Seoul National University Boramae Hospital Seoul Republic of Korea; ^5^ Department of Internal Medicine Seoul National University College of Medicine Seoul Republic of Korea; ^6^ Department of Internal Medicine Seoul National University Bundang Hospital Seongnam Republic of Korea

**Keywords:** ageing, biomarkers, extracellular vesicle, sarcopenia, skeletal muscle

## Abstract

**Background:**

As society ages, identifying individuals at risk of sarcopenia becomes essential. Several plasma biomarkers are used to assess musculoskeletal status, but their results are inconsistent. Extracellular vesicles (EVs) are investigated as disease biomarkers due to their role in transporting molecules and influencing cellular processes. This study investigated the correlation of known sarcopenia biomarkers—adiponectin, myostatin, P3NP, CRP and TNF‐α—measured from plasma‐derived EVs with muscle mass, function and performance in an Osteoporosis Sarcopenia cohort at the Seoul National University Bundang Hospital.

**Methods:**

Muscle mass was evaluated by measuring appendicular skeletal muscle mass (ASM) using dual X‐ray absorptiometry and calculated as ASM/height^2^. Hand grip strength was measured using a hydraulic hand dynamometer for muscle function and physical performance based on the Short Physical Performance Battery (SPPB), walking speed and the five‐time‐sit‐to‐stand test. Density gradient ultracentrifugation was used to isolate EVs from the plasma, followed by confirming the expression of sarcopenia biomarkers. Multivariate regression analysis, adjusted for sex, age, body mass index, smoking, drinking, and bone density, was performed.

**Results:**

The mean age of participants was 74.3 ± 12.1 years (range, 52.0–96.0), with 88.2% being female. Plasma‐derived EV levels of myostatin and P3NP were significantly associated with walking speed (*ꞵ* = −0.309, *p* = 0.014) and SPPB (*ꞵ* = −0.276, *p* = 0.029), respectively. TNF‐α levels were strongly correlated with hand grip strength (*ꞵ* = −0.313, *p* = 0.013). Using receiver‐operating characteristic curve analysis, cutoff values for three factors were determined, allowing participants to be categorized into high and low groups. Low myostatin group had a higher hand grip strength (19.63 kg vs. 17.14 kg, *p* = 0.027) and faster five‐time‐sit‐to‐stand test times (17.34 s vs. 23.72 s, *p =* 0.032). Low P3NP levels showed a stronger grip strength (19.87 kg vs. 16.81 kg, *p* = 0.008), better SPPB scores (9.10 vs. 8.03, *p =* 0.006) and five‐time‐sit‐to‐stand times (18.31 s vs. 21.87 s, *p* = 0.002). Low TNF‐α levels were linked to better walking speeds (0.82 m/s vs. 0.64 m/s, *p* = 0.009) and lower SARC‐F scores (1.73 vs. 3.26, *p* = 0.029).

**Conclusion:**

Our research confirmed that EVs‐derived myostatin, P3NP and TNF‐α are strongly associated with muscle function and performance. Significant differences in these factors between high and low groups based on biomarker cutoffs emphasize their diagnostic relevance for sarcopenia. These findings offer a promising avenue for identifying effective markers in future research and clinical applications.

## 1 Introduction

Sarcopenia, characterized by the age‐related decline in skeletal muscle mass, strength and physical performance [1], is a significant predictor of various adverse health outcomes, including elevated risk of falls and fractures [2], cardiovascular diseases [3], respiratory disorders [4] and cognitive impairment [5]. Its prevalence increases dramatically with age, affecting approximately 10% of individuals aged 60–69 years and more than 40% of those over 80 years [6]. Sarcopenia is linked to increased all‐cause mortality and represents a leading cause of disability, morbidity and mortality among older adults, contributing to higher medical costs. Therefore, early diagnosis and accurate prognosis prediction are crucial to managing this condition effectively [7].

Various international groups, including the European Working Group on Sarcopenia in Older People (EWGSOP), the Asian Working Group for Sarcopenia (AWGS) and the Sarcopenia Definitions and Outcomes Consortium (SDOC), supported by the National Institute on Aging and the Foundation for the National Institutes of Health, have established diagnostic criteria for the disease [1, 8, 9]. These guidelines predominantly rely on physical parameters such as muscle mass, function and physical performance. However, these methods present challenges related to repeatability, accessibility and practicality within the geriatric population. The development and validation of a panel of multiple biomarkers, including blood‐based markers, could provide specific, noninvasive and cost‐effective tests for diagnosing and monitoring sarcopenia in diverse clinical settings.

Despite ongoing efforts, the lack of consensus on sarcopenia biomarkers hinders progress in diagnostic and treatment. The Expert Group Meeting Organized by the European Society for Clinical and Economic Aspects of Osteoporosis, Osteoarthritis and Musculoskeletal Diseases (ESCEO) [10] has recommended specific biomarkers for analysis in clinical trials addressing sarcopenia. Our study aligns with these recommendations by focusing on biomarkers categorized based on their functions into groups, including adiponectin, myostatin, procollagen type 3 N‐terminal peptide (P3NP), C‐reactive protein (CRP) and tumour necrosis factor‐alpha (TNF‐α).

Extracellular vesicles (EVs) are membrane‐enclosed particles released into the extracellular milieu, including the bloodstream, by most cell populations. Protected by a lipid bilayer membrane, EVs carry proteins, metabolites, lipids and nucleic acids, ensuring cargo stability, supporting intercellular communication and improving diagnostic accuracy [11]. Their presence in accessible biological fluids such as urine, plasma and serum highlights their potential as noninvasive biomarkers for a range of diseases [12]. Although EVs have been extensively studied in musculoskeletal conditions such as osteoarthritis [13], their relationship with sarcopenia diagnostic criteria remains underexplored, particularly in diverse human cohorts. This gap underscores the importance of our study, which aims to provide a more comprehensive understanding of how EVs‐derived biomarkers correlate with muscle mass, function and performance in sarcopenia.

This study aimed to examine the association of biomarkers recommended by the ESCEO guidelines—including adiponectin, myostatin, P3NP, CRP and TNF‐α—with sarcopenia diagnostic criteria using plasma‐derived EV markers in a prospective cohort. Additionally, this study sought to validate these biomarkers within a single cohort, contributing to the development of reliable biomarkers and therapeutic strategies that enhance early detection and treatments for sarcopenia.

## 2 Materials and Methods

### 2.1 Study Design and Participants

Participants for this study were enrolled from the Osteoporosis Sarcopenia (OsteoSarc) cohort at Seoul National University Bundang Hospital (SNUBH), Korea, which was initiated in 2021. By June 2024, the cohort included 526 participants and is still open for recruitment. The OsteoSarc cohort is an ongoing prospective study collecting baseline and annual measurements of demographics, bone mineral density (BMD), muscle mass, muscle function and physical performance. The inclusion criteria of the cohort were adults aged ≥ 50 years with a BMD T‐score of ≤ −2.5 at the lumbar spine, femoral neck or total hip and adults with a history of osteoporotic fractures. Because all patients could walk on their own in this study, we mainly evaluated the physical performance based on Short Physical Performance Battery (SPPB), walking speed and the five‐time‐sit‐to‐stand test. Subjects without osteoporosis and those with metabolic bone diseases or active cancer were excluded from the study. Among the participants, the analysis included 93 participants followed up for 2 years. Among these subjects, 54 patients were diagnosed with sarcopenia according to the criteria of the AWGS [9]. The Institutional Review Board of Seoul National University Bundang Hospital approved this study (No. B‐2104‐678‐302). Informed written consent was taken from all study participants according to Helsinki's declaration.

### 2.2 Demographic Characteristics

Clinical data, including age, sex, drinking and smoking habits, history of fractures and comorbidities (such as diabetes, hypertension and rheumatoid arthritis), were obtained from hospital information systems and health interviews. A history of smoking is an important predictor of sarcopenia onset [14]. Participants were classified as ever smokers if they had smoked > 5 packs of cigarettes in their lifetime or had quit smoking at least 6 months before the baseline. Ever drinkers were defined as those consuming > 5 g of ethanol daily or had quit drinking at least 6 months before the baseline. Height and body weight were measured to the nearest 0.1 cm and 0.1 kg, respectively, with participants wearing light garments. Body mass index (BMI) was calculated by dividing body weight by height squared (kg/m^2^). Trained staff collected all data using standardized protocols.

### 2.3 Assessment of Sarcopenia

**FIGURE 1:**
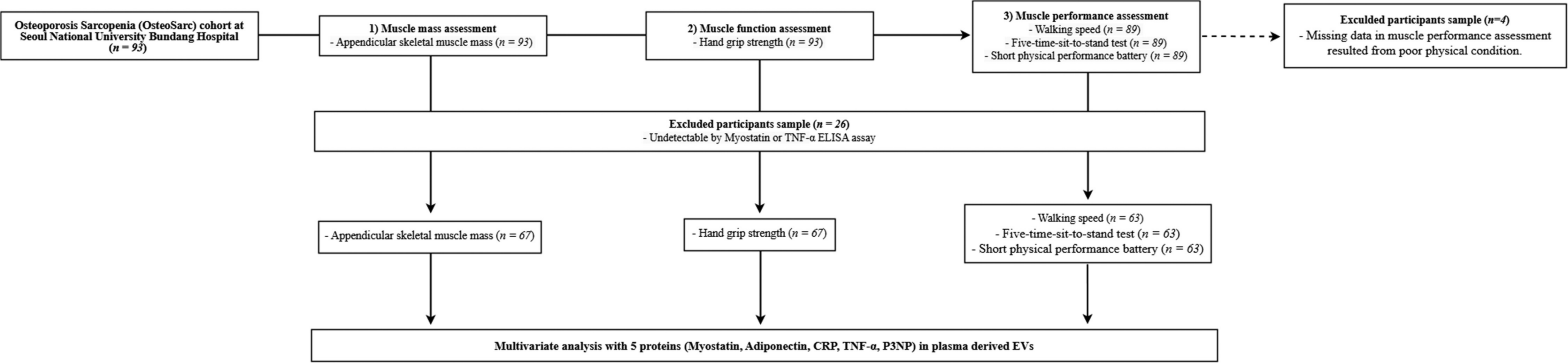
Flow chart of the study design. CRP, C‐reactive protein; P3NP, procollagen type 3 N‐terminal peptide; TNF‐α, tumour necrosis factor‐alpha.

Muscle mass was evaluated by measuring appendicular skeletal muscle mass (ASM) using dual X‐ray absorptiometry (Discovery W; Hologic, United States) and calculated as ASM/height^2^ (m^2^). Hand grip strength was measured using a hydraulic hand dynamometer (grip strength dynamometer 5401 Grip‐D; Takei Scientific Instruments Co., Ltd., Japan). The tests were performed three times with each hand with 60 s rest between each trial, and the highest value was recorded for analysis. Physical performance was assessed using the SPPB score comprising three timed tests: balance, 4‐m walking speed and the five‐time‐sit‐to‐stand test, with higher scores indicating better performance. In the balance test, participants attempted to hold the side‐by‐side, semitandem and tandem positions for 10 s [15]. Walking speed was evaluated by measuring the participant's usual gait speed (m/s) over a 4‐m course, recording the fastest of two attempts for analysis. For the five‐time‐sit‐to‐stand test, participants were asked to stand up from a chair with their arms folded across the chest five times in a row as quickly as possible, and the time required to complete the task was recorded. Assistive devices were permitted if necessary. Among the 93 participants, four individuals were unable to perform muscle performance tests, including walking speed and the five‐time‐sit‐to‐stand test, due to poor physical condition. Because the SPPB score is calculated using results from these tests, their SPPB scores were also unavailable (Figure 1). Participants were categorized into high and low sarcopenia‐related factor groups based on thresholds established by the Asian Working Group for Sarcopenia (AWGS) and the Korean Working Group for Sarcopenia (KWGS) guidelines [9, 16]. Walking speed was classified using a cutoff of < 1 m/s. The strength, assistance with walking, rising from a chair, climbing stairs and falls (SARC‐F) score was split at ≥ 4. Hand grip strength thresholds were < 28 kg for males and < 18 kg for females. The five‐time‐sit‐to‐stand test was classified using a cutoff of ≤ 12 s. Lastly, the SPPB score was used, with a score of ≤ 9.

### 2.4 Plasma EV Isolation

Human blood was extracted from each donor by aseptic venipuncture and collected into a vacuum tube (5 mL) containing EDTA as an anticoagulant. Plasma was obtained by centrifuging whole blood at room temperature for 10 min at 10 000 g and stored at −80°C. Plasma EVs were isolated by differential gradient ultracentrifugation (DGUC) as previously described by Onodi et al. [17]. Briefly, the Optiprep solution (#07820, STEMCELL, Canada) was diluted to 50%, 30% and 10% concentrations using a 0.25‐M sucrose buffer (0.25‐M sucrose, 1‐mM EDTA and 10‐mM Tris–HCl, pH 7.4). In a 3.7‐mL tube (329561A, Himac, Germany), 1.17 mL of each diluted solution was layered sequentially to establish a discontinuous gradient. Then, the prepared plasma was layered onto these gradients. The samples were subsequently centrifuged for 24 h at 120 000×*g* at 4°C using the P40ST rotor in an ultracentrifuge (CP100WX, Himac, Germany). Following centrifugation, 10 fractions were sequentially collected from the top. Using DGUC, plasma lipoproteins were depleted, thereby enhancing the purity of the EVs sample. All fractions were stored at −80°C for future analysis. The yield of plasma EVs was assessed by quantifying the total protein content using the Bicinchoninic Acid (BCA) Kit (#23225, Thermo Scientific, United States). The sample absorbance was read at 562 nm using a SpectraMax i3x multimode microplate reader (Molecular Devices, United States). Generally, 345.6 μg of the total protein content of EVs could be obtained from 0.2 mL of plasma.

### 2.5 Abundant Proteins Depletion in EVs

The Multiple Affinity Removal Spin Cartridge Human‐6 (#5188‐5230, Agilent, United States) was utilized to deplete six highly abundant proteins (albumin, IgG, IgA, transferrin, haptoglobin and antitrypsin) from EV samples, following the manufacturer's protocol.

### 2.6 Nanoparticle Tracking Analysis and Transmission Electron Microscopy

The size and concentration of plasma EVs were analysed using a NanoSight LM10 system (Malvern Panalytical, United Kingdom) as previously described [18]. The analysis utilized the NTA 2.3 software, with 60‐s videos captured for each sample in triplicate. The camera level was adjusted to 15–16, and the detection threshold was set at 10. The morphology and size of EVs were also examined using a Hitachi TEM system (Hitachi, HT7700) as previously described [18].

### 2.7 Western Blot Analysis

The EVs‐enriched fractions (56.2 μL) were supplemented with 4X LDS (B0007, Thermo Scientific, United States) and subjected to western blotting for the classical EVs markers (CD9, 25 kD; CD63, 40–60 kD), along with the major plasma protein ApoB (512 kD) and ApoE (36 kDa), to analyse the purity and protein contents of EVs. Briefly, 30 μL of each sample was loaded onto a Bolt 4%–12% Bis‐Tris gel (NW04120BOX, Invitrogen, United States) and transferred to a polyvinylidene difluoride (PVDF) membrane. The membrane was stained with Ponceau‐S (A40000279, Thermo Scientific, United States) for 2 min to assess transfer efficiency and visualize total protein in each lane. After confirming the transfer, the membrane was rinsed with methanol and then blocked in 5% bovine serum albumin in tris‐buffered saline with Tween 20 detergent (TBST; #28358, Thermo Scientific, United States) at room temperature for 1.5 h. Primary antibodies were diluted in the blocking solution and incubated with the membranes at 4°C. The primary antibodies used were anti‐CD9 (1:1000; GTX76185, GeneTex, United States), anti‐CD63 (1:1000; sc‐5275, Santa Cruz Biotechnology, United States), anti‐ApoB (1:1000; sc‐13538, Santa Cruz Biotechnology, United States), anti‐ApoE (1:1000; sc‐390 925, Santa Cruz Biotechnology, United States), anti‐Albumin (1:1000; sc‐271605, Santa Cruz Biotechnology, United States) and anti‐IgG (1:1000; sc‐515946, Santa Cruz Biotechnology, United States). The membrane was washed for 5 min three times in a TBST buffer, followed by incubation with a horseradish peroxidase–conjugated secondary antimouse antibody (1:10000; #7076, Cell Signaling Technology) at room temperature for 1.5 h. Band detection was performed using the SuperSignal West Femto Maximum Sensitivity Substrate (#34094, Thermo Scientific, United States) in a ChemiDoc Touch Imaging System (Bio‐Rad, United States). Band intensity was quantified using Image Lab software (Bio‐Rad, United States).

### 2.8 Measurement of Sarcopenia Biomarkers

After excluding samples with undetectable protein levels by enzyme‐linked immunosorbent assay (ELISA) and missing sarcopenia criteria values, the analysis included 67 samples for muscle mass and muscle function and 63 samples for walking speed, SPPB and five‐time‐sit‐to‐stand test (Figure 1). The levels of P3NP (OKEH00548, Aviva System Biology, United States), TNF‐ α (HSTA00E, R&D systems, United States), myostatin (DGDF80, R&D systems, United States), adiponectin (EZHADP‐61 K, Millipore, United States) and CRP (ab99995, Abcam, United Kingdom) in the pooled EVs‐enriched fractions (Fractions 5–7) isolated from the plasma were measured using ELISA.

### 2.9 Statistical Analysis

Univariate and multivariate linear regressions were performed to evaluate the correlation of each biomarker with sarcopenia criteria, adjusting for age, sex, BMI, smoking, drinking and femoral neck BMD. The independent variables were standardized to mean = 0 and standard deviation = 1 before calculating the standardized coefficient beta in the regression model. All analyses were conducted using R statistical software (Version 4.2.1).

## 3 Results

### 3.1 Baseline Features of the Study Population

Table 1 summarizes the baseline characteristics and measured concentrations of the sarcopenia markers in plasma‐derived EVs of participants (*n* = 93) from the OsteoSarc prospective cohort from Seoul National University Bundang Hospital. This study cohort included 82 (88.2%) females, with a mean age of 74.3 ± 12.1 (range 52.0–96.0) years and a mean BMI of 22.2 ± 4.2 (range 11.0–43.3). For sarcopenia‐related factors, participants had a mean ASM/height^2^ of 4.93 ± 0.80 kg/m^2^, a mean hand grip strength of 18.78 ± 5.94 kg, a mean walking speed of 0.79 ± 0.23 m/s, a mean SPPB score of 8.76 ± 2.45 and an average time of 19.55 ± 14.20 s for the five‐time‐sit‐to‐stand test. The SARC‐F score, commonly used to screen individuals at risk for sarcopenia, had an average score of 2.05 ± 2.11 [16]. Additionally, the participants' BMD showed an average lumbar spine BMD (L1–4) of 0.748 ± 0.127 g/cm^2^, with femoral neck BMD and total hip BMD measuring 0.522 ± 0.084 g/cm^2^ and 0.656 ± 0.093 g/cm^2^, respectively.

**TABLE 1:** Baseline characteristics of the study population.

Variable	Mean ± SD	Median (IQR)
Gender, female *n* (%)	82 (88.2)	76 (22.00)
Age, years	74.31 ± 12.09	21.48 (4.35)
Body mass index, kg/m^2^	22.15 ± 4.19	21.50 (4.24)
History of fracture, *n*	33 (35.4%)	—
History of fall, *n*	9 (9.6%)	—
Smoking, *n*	8 (8.6%)	—
Drinking, *n*	21 (22.5%)	—
Family history of fracture, *n*	38 (40.8%)	—
Diabetes mellitus, *n*	9 (9.7%)	—
Hypertension, *n*	32 (34.4%)	—
Rheumatoid arthritis, *n*	4 (4.3%)	
ADL, score	20.52 ± 1.82	21 (0.00)
IADL, score	25.09 ± 3.86	27 (1.00)
K‐MMSE, score	11.40 ± 6.86	15 (7.50)
SARC‐F, score	2.05 ± 2.11	1.0 (3.00)
Lumbar spine BMD (L1–4) (g/cm^2^)	0.748 ± 0.127	0.718 (0.158)
Femoral neck BMD (g/cm^2^)	0.522 ± 0.084	0.516 (0.113)
Total Hip BMD (g/cm^2^)	0.656 ± 0.093	0.651 (0.139)
Sarcopenia, *n* (%)	54 (60.6)	—
Appendicular skeletal muscle mass/height^2^, kg/m^2^	4.93 ± 0.80	4.87 (0.86)
Hand grip strength, kg	18.78 ± 5.94	18.01 (6.80)
Walking speed, m/s	0.79 ± 0.23	0.80 (0.33)
Short physical performance battery, score	8.76 ± 2.45	9.00 (3.00)
Five‐time‐sit‐to‐stand test, s	19.55 ± 14.20	15.00 (10.00)
Sarcopenia markers		
Myostatin, pg/mL	20.21 ± 23.85	15.99 (16.71)
P3NP, ng/mL	0.49 ± 0.12	0.48 (0.10)
Adiponectin, ng/mL	306.29 ± 95.59	325.86 (134.00)
CRP, ng/mL	1.46 ± 1.19	1.19 (2.05)
TNF‐α, pg/mL	0.14 ± 0.15	0.11 (0.16)

*Note:* All of ± data indicate mean ± standard deviation.

Abbreviations: ADL, activities of daily living; BMD, bone mineral density; IADL, instrumental activities of daily living; K‐MMSE, Korean mini mental state exam; P3NP, procollagen 3 N‐terminal peptide; SARC‐F, strength, assistance with walking, rising from a chair, climbing stairs and falls.

Participants were divided into normal (*n* = 35) and sarcopenia (*n* = 54) groups based on the AWGS diagnostic criteria (Table 2) [16]. When participants were grouped based on sarcopenia status, 35 participants (39.3%) were classified as participants without sarcopenia, whereas 54 participants (60.7%) were classified as participants with sarcopenia. Among the normal group, the mean age was 72.4 ± 13.0 years, with a BMI of 22.6 ± 4.7 kg/m^2^, whereas the sarcopenia group had a mean age of 75.5 ± 11.7 years and BMI of 21.8 ± 4.0 kg/m^2^. These differences were not statistically significant.

**TABLE 2:** Baseline characteristics of the normal and sarcopenia participant.

Variable	Normal (*n* = 35)	Sarcopenia (*n* = 54)	*p* value
Gender, female *n* (%)*	**35 (39.3%)**	**43 (60.7%)**	**0.004**
Age, years	72.42 ± 13.03	75.46 ± 11.70	0.255
Body mass index, kg/m^2^	22.60 ± 4.69	21.81 ± 3.99	0.545
History of fracture, n*	9 (10.1%)	24 (27.0%)	0.074
History of fall, n*	3 (3.4%)	6 (6.7%)	0.698
Smoking, n*	2 (2.2%)	5 (5.6%)	0.544
Drinking, n*	11 (12.4%)	9 (12.1%)	0.161
Family history of fracture, n*	17 (19.1%)	21 (23.6%)	0.367
Diabetes mellitus, n*	4 (4.5%)	5 (5.6%)	0.740
Hypertension, n*	12 (13.5%)	19 (21.3%)	0.931
Rheumatoid arthritis, n*	2 (2.2%)	2 (2.2%)	0.655
ADL, score	20.97 ± 0.16	20.37 ± 2.27	0.097
IADL, score	**26.57 ± 1.42**	**24.57 ± 4.43**	**0.006**
K‐MMSE, score	12.17 ± 6.87	11.53 ± 6.61	0.288
SARC‐F, score	**1.05 ± 1.08**	**2.29 ± 2.01**	**0.002**
Lumbar spine BMD (L1–4) (g/cm^2^)	**0.753 ± 0.120**	**0.748 ± 0.133**	**0.861**
Femoral neck BMD (g/cm^2^)	**0.558 ± 0.080**	**0.505 ± 0.080**	**0.003**
Total Hip BMD (g/cm^2^)	**0.688 ± 0.073**	**0.645 ± 0.097**	**0.023**
Appendicular skeletal muscle mass/height^2^, kg/m^2^	**5.29 ± 0.97**	**4.68 ± 0.58**	**0.001**
Hand grip strength, kg	**22.97 ± 5.80**	**16.57 ± 4.23**	**< 0.001**
Walking speed, m/s	**0.88 ± 0.16**	**0.73 ± 0.25**	**0.004**
Short physical performance battery, score	**10.26 ± 1.37**	**7.75 ± 2.48**	**< 0.001**
Five‐time‐sit‐to‐stand test, s	**13.00 ± 4.73**	**23.80 ± 16.54**	**< 0.001**

*Note:* All of ± data indicate mean ± standard deviation. *p* values were calculated using the Mann–Whitney test and *Chi‐square test. Statistically significant results are highlighted in bold (*p* < 0.05).

Abbreviations: ADL, activities of daily living; BMD, bone mineral density; IADL, instrumental activities of daily living; K‐MMSE, Korean mini mental state exam; SARC‐F, strength, assistance with walking, rising from a chair, climbing stairs and falls.

Muscle mass, measured as ASM/height^2^, was significantly higher in the normal group (5.29 ± 0.97 kg/m^2^) compared to the sarcopenia group (4.68 ± 0.58 kg/m^2^, *p* = 0.001). Similarly, hand grip strength was significantly greater in the normal group (22.97 ± 5.80 kg) than in the sarcopenia group (16.57 ± 4.23 kg, *p* < 0.001). Walking speed was also significantly slower in the sarcopenia group (0.73 ± 0.25 m/s) than in the normal group (0.88 ± 0.16 m/s, *p* = 0.004).

Performance metrics, including the SPPB score and five‐time‐sit‐to‐stand test, showed significant differences between groups. The SARC‐F score was significantly higher in the sarcopenia group, with a value of 2.29 ± 2.01 compared to 1.05 ± 1.08 in the normal group (*p* = 0.002). Additionally, lumbar spine, femoral neck and total hip BMD values were lower in the sarcopenia group than in the normal group, consistent with expected patterns in sarcopenia‐related comorbidities.

### 3.2 Isolation of Plasma EVs

EVs were isolated by DGUC through a multistep density‐gradient overlay. Equal volume fractions (F1–10) were collected sequentially from the top of the tube, enabling the removal of major impurities, such as lipoproteins (Figure S1A) [17]. Immunoblot staining for canonical EV markers (CD9 and CD63) showed enrichment in Fractions 5–7, whereas Apolipoproteins B and E were successfully depleted (Figure S1B). To assess potential contamination of plasma‐abundant proteins, we examined the presence of albumin and IgG in three additional plasma‐derived EV samples and attempted to deplete them. Although commercially available albumin and IgG depletion kits effectively reduced these proteins, this process also resulted in a significant loss of EVs, as indicated by reduced CD9 levels (Figure S1C). Considering previous studies reporting that albumin and IgG are intrinsic components of plasma‐derived EVs rather than contaminants, we proceeded to collect EV samples without performing albumin and IgG depletion [19]. Transmission electron microscopy confirmed the presence of typical EVs with diameters ranging from 30 to 200 nm in these fractions (Figure S1D). Nanoparticle‐tracking analyses revealed average modal size of 186 ± 27 nm, 204 ± 16 nm and 215 ± 13 nm in Fractions 5–7, respectively, consistent with the known size range of EVs (Figure S1E) [20]. Average particle concentrations were 14.8 × 10^8^ particles/mL, 16 × 10^8^ particles/mL and 30 × 10^8^ particles/mL in Fractions 5–7, respectively, with an average recovery of 5.95 × 10^9^ particles/mL of plasma input. Ponceau‐S staining and BCA assay indicated reduced soluble plasma protein contamination in these EV‐enriched fractions compared to earlier fractions (Figure S1B,F). Thus, Fractions 5–7 were confirmed as high‐purity EVs‐enriched fractions and subsequently used to quantify sarcopenia‐associated markers, including adiponectin, myostatin, P3NP, CRP and TNF‐α, within plasma‐derived EVs using ELISA.

### 3.3 Associations Between Plasma‐Derived EV Markers and Sarcopenia Diagnostic Criteria

Tables 3, 4, 5 present multiple regression models evaluating the associations between sarcopenia markers (adiponectin, myostatin, P3NP, CRP and TNF‐α) and sarcopenia criteria, including ASM/height^2^ (Table 3), hand grip strength (Table 4) and performance (walking speed, SPPB and the five‐time‐sit‐to‐stand test) (Table 5). Significant associations were observed for several biomarkers. Myostatin levels were negatively associated with walking speed (*ꞵ* = −0.309, *p* = 0.014), whereas P3NP levels were negatively associated with SPPB scores (*ꞵ* = −0.276, *p* = 0.029). TNF‐α levels showed a strong negative correlation with hand grip strength (*ꞵ* = −0.313, *p* = 0.013). These associations remained significant after adjusting for potential confounders, including sex, age, BMI, smoking, drinking and femoral neck BMD.

**TABLE 3:** Unadjusted and adjusted associations between markers in plasma‐derived EVs and ASM/height^2^.

	Univariate		Multivariate	
	*ꞵ*	*p* value	*ꞵ*	*p* value
Myokines				
Myostatin	−0.219	0.075	−0.119	0.277
Others				
P3NP	0.119	0.338	0.143	0.180
Adipokines				
Adiponectin	0.060	0.632	0.111	0.316
Inflammatory markers				
CRP	−0.092	0.461	0.045	0.678
TNF‐α	−0.151	0.222	−0.035	0.749

Abbreviations: *ꞵ*, standardized coefficient beta calculated by univariate and multivariable regression analysis adjusted for age, sex, BMI, smoking, drinking and femoral neck (g/cm^2^); CRP, C‐reactive protein; P3NP, procollagen type 3 N‐terminal peptide; TNF‐α, tumour necrosis factor‐alpha.

**TABLE 4:** Unadjusted and adjusted associations between markers in plasma‐derived EVs and hand grip strength.

	Univariate		Multivariate	
	*ꞵ*	*p* value	*ꞵ*	*p* value
Myokines				
Myostatin	−0.148	0.232	−0.090	0.481
Others				
P3NP	−0.130	0.294	−0.086	0.491
Adipokines				
Adiponectin	−0.022	0.858	0.053	0.684
Inflammatory markers				
CRP	0.151	0.223	0.138	0.280
TNF‐α	−**0.295**	**0.015**	**−0.313**	**0.013**

*Note:* Statistically significant results are highlighted in bold (*p* < 0.05).

Abbreviations: *ꞵ*, standardized coefficient beta calculated by univariate and multivariable regression analysis adjusted for age, sex, BMI, smoking, drinking and femoral neck (g/cm^2^); CRP, C‐reactive protein; P3NP, procollagen type 3 N‐terminal peptide; TNF‐α, tumour necrosis factor‐alpha.

**TABLE 5:** Unadjusted and adjusted associations between markers in plasma‐derived EVs and muscle performance (walking speed, SPPB and the five‐time‐sit‐to‐stand test).

	Walking speed				SPPB				Five‐time‐sit‐to‐stand test			
	Univariate		Multivariate		Univariate		Multivariate		Univariate		Multivariate	
	*ꞵ*	*p* value	*ꞵ*	*p* value	*ꞵ*	*p* value	*ꞵ*	*p* value	*ꞵ*	*p* value	*ꞵ*	*p* value
Myokines												
Myostatin	−**0.337**	**0.007**	**−0.309**	**0.014**	−0.121	0.347	−0.109	0.407	**0.262**	**0.038**	0.209	0.093
Others												
P3NP	−0.188	0.141	−0.145	0.250	−**0.279**	**0.027**	**−0.276**	**0.029**	0.115	0.368	0.085	0.494
Adipokines												
Adiponectin	−0.045	0.724	0.026	0.846	−0.045	0.727	0.047	0.727	0.055	0.668	−0.058	0.658
Inflammatory markers												
CRP	0.133	0.298	0.071	0.588	0.144	0.261	0.093	0.481	−0.021	0.872	−0.025	0.847
TNF‐α	−0.163	0.203	−0.174	0.187	−0.102	0.427	−0.090	0.502	**0.258**	**0.041**	0.231	0.070

*Note:* Statistically significant results are highlighted in bold (*p* < 0.05).

Abbreviations: *ꞵ*, standardized coefficient beta calculated by univariate and multivariable regression analysis adjusted for age, sex, BMI, smoking, drinking and femoral neck (g/cm^2^); CRP, C‐reactive protein; P3NP, procollagen type 3 N‐terminal peptide; SPPB, short physical performance battery; TNF‐α, tumour necrosis factor‐alpha.

To further explore these relationships, ROC curve analysis was performed to determine optimal cutoff values for the biomarkers. The thresholds identified were 21.234 pg/mL for myostatin, 0.514 ng/mL for P3NP and 0.188 pg/mL for TNF‐α. Participants were categorized into high and low groups based on these thresholds, and significant differences in sarcopenia‐related factors were observed (Table 6).

**TABLE 6:** Comparison of sarcopenia related factors between low and high groups based on myostatin, P3NP and TNF‐α cutoff values.

	Myostatin cutoff value: 21.234 pg/mL			P3NP cutoff value: 0.514 ng/mL			TNF‐α cutoff value: 0.188 pg/mL		
	Total (*n* = 82)		*p* value	Total (*n* = 93)		*p* value	Total (*n* = 72)		*p* value
	Low (*n* = 53)	High (*n* = 29)		Low (*n* = 60)	High (*n* = 33)		Low (*n* = 53)	High (*n* = 19)	
SARC‐F, score	1.62 ± 1.48	2.79 ± 2.96	0.282	1.86 ± 2.21	2.39 ± 1.93	0.052	**1.73 ± 1.87**	**3.26 ± 2.86**	**0.029**
ASM/height^2^, kg/m^2^	4.95 ± 0.96	4.82 ± 0.53	0.551	5.03 ± 0.88	4.73 ± 0.58	0.117	4.96 ± 0.91	4.69 ± 0.67	0.184
Hand grip strength, kg	**19.63 ± 5.88**	**17.14 ± 5.70**	**0.027**	**19.87 ± 6.53**	**16.81 ± 4.09**	**0.008**	18.70 ± 6.03	16.99 ± 5.87	0.138
Walking speed, m/s	0.81 ± 0.22	0.77 ± 0.26	0.472	0.81 ± 0.22	0.76 ± 0.25	0.316	**0.82 ± 0.23**	**0.64 ± 0.22**	**0.009**
SPPB, score	9.30 ± 2.18	8.20 ± 2.44	0.052	**9.10 ± 2.65**	**8.03 ± 1.87**	**0.006**	8.98 ± 2.46	7.75 ± 2.64	0.083
Five‐time‐sit‐to‐stand test, s	**17.34 ± 12.45**	**23.72 ± 17.10**	**0.032**	**18.31 ± 15.08**	**21.87 ± 12.28**	**0.002**	20.13 ± 15.80	19.07 ± 12.09	0.481

*Note:* *p* values were calculated using the Mann–Whitney test. Statistically significant results are highlighted in bold (*p* < 0.05).

Abbreviations: ASM, appendicular skeletal muscle mass; P3NP, procollagen type 3 N‐terminal peptide; SPPB, short physical performance battery; TNF‐α, tumour necrosis factor‐alpha.

For myostatin, participants in the low group demonstrated better muscle function, with higher hand grip strength (19.63 ± 5.88 kg in the low group vs. 17.14 ± 5.70 kg in the high group, *p* = 0.027) and faster five‐time‐sit‐to‐stand test times (17.34 ± 12.45 s in the low group vs. 23.72 ± 17.10 s in the high group, *p* = 0.032). Similarly, participants in the low P3NP group exhibited superior muscle function and performance, with higher hand grip strength (19.87 ± 6.53 kg in the low group vs. 16.81 ± 4.09 kg in the high group, *p* = 0.008), higher SPPB scores (9.10 ± 2.65 in the low group vs. 8.03 ± 1.87 in the high group, *p* = 0.006) and shorter the five‐time‐sit‐to‐stand test (18.31 ± 15.08 s in the low group vs. 21.87 ± 12.28 s in the high group, *p* = 0.002).

For TNF‐α, participants in the low group had significantly better physical performance, with faster walking speeds (0.82 ± 0.23 m/s in the low group vs. 0.64 ± 0.22 m/s in the high group, *p* = 0.009) and lower SARC‐F scores (1.73 ± 1.87 in the low group vs. 3.26 ± 2.86 in the high group, *p* = 0.029). These findings are consistent with our multivariate analysis results (Tables 4 and 5), indicating that myostatin, P3NP and TNF‐α are more closely associated with muscle function and performance than muscle mass.

When comparing EV biomarkers' concentration between the normal and sarcopenia groups, no significant differences were observed when comparing the concentrations of myostatin, P3NP and TNF‐α. Specifically, the normal group had a myostatin level of 15.14 ± 11.79 pg/mL, whereas the sarcopenia group had 22.42 ± 29.31 pg/mL (*p* = 0.153). P3NP levels were 0.47 ± 0.06 ng/mL in the normal group and 0.49 ± 0.07 ng/mL in the sarcopenia group (*p* = 0.131). Similarly, TNF‐α levels were 0.11 ± 0.08 pg/mL in the normal group and 0.15 ± 0.17 pg/mL in the sarcopenia group (*p* = 0.597).

Further analyses categorized participants based on the cutoff values of established sarcopenia‐related factors to assess whether EV biomarkers showed significant differences across groups [16] (Table S1). Consistent with previous results, myostatin concentrations were significantly higher in the high SARC‐F score group (35.6 ± 47.7 pg/mL) compared to the low SARC‐F group (16.7 ± 12.2 pg/mL, *p* = 0.040). Additionally, CRP levels were significantly higher in the high SARC‐F group (2.1 ± 0.1 μg/mL) compared to the low SARC‐F group (1.3 ± 0.1 μg/mL, *p* = 0.004).

## 4 Discussion

This study investigated the relationship between EVs‐derived sarcopenia markers and various sarcopenia‐related factors using the OsteoSarcopenia cohort. Among the factors examined, muscle function and muscle performance—including walking speed, SPPB and the five‐time‐sit‐to‐stand test—exhibited strong correlations with biomarkers present in EVs. After adjusting for age, sex, BMI, smoking, drinking and femoral neck BMD, TNF‐α was associated with muscle function, myostatin correlated with walking speed and P3NP lined to the SPPB scores. Although no significant differences in EV biomarker concentrations were observed between participants classified as sarcopenic or nonsarcopenic, ROC curve‐derived cutoff values for myostatin, P3NP and TNF‐α revealed significant group differences in functional sarcopenia parameters, including SARC‐F score, hand grip strength, walking speed and five‐time‐sit‐to‐stand test. These findings suggest that EV biomarkers may be particularly effective in assessing muscle function and performance impairments associated with sarcopenia.

Interestingly, our findings reveal a significant correlation between EV biomarkers and muscle function and performance rather than muscle mass, aligning with prior studies [21]. For example, the EXERNET‐Elder 3.0 project identified PF4 and C1R as potential EV biomarkers for sarcopenia, with strong associations to muscle function and physical performance but not muscle mass [21]. Moreover, prior research has consistently reported that muscle function and performance often do not directly correlate with muscle mass [22]. These findings underscore the potential of EV biomarkers to reflect muscle function and performance more effectively than muscle mass.

Within the muscular system, EVs are actively secreted by myoblasts and carry factors critical for muscle repair, such as insulin‐like growth factor‐1 (IGF‐1) [23] and fibroblast growth factor (bFGF) [24], promoting muscle regeneration. Conversely, EVs containing factors like growth differentiation factor 15 (GDF‐15) [25] and interleukin‐6 (IL‐6) [26] contribute to muscle wasting. Although the underlying molecular mechanisms remain to be fully elucidated, these studies underscore the relevance of EVs in the pathophysiology of skeletal muscle disorders, reinforcing their potential as biomarkers for sarcopenia. Furthermore, plasma proteins and EV proteins exhibit distinct expression patterns. For example, studies using plasma samples have reported an average correlation coefficient of approximately 0.187 for biomarkers candidates like myostatin, P3NP and TNF‐α with muscle parameters [27, 28, 29, 30]. Although differences in cohort characteristics and statistical methods may influence these results, our findings revealed stronger correlations with EV biomarkers, with an average coefficient of 0.299, despite accounting for a greater number of variables in our multivariable analysis. These results suggest that EV biomarkers may provide more accurate and reliable associations with muscle‐related parameters compared to plasma biomarkers.

Among the markers studied, TNF‐α, the inflammatory marker, was negatively correlated with muscle function and positively correlated with the five‐time‐sit‐to‐stand test. These findings are consistent with previous studies demonstrating that an inverse association of TNF‐α with hand grip strength [28]. TNF‐α contributes to muscle protein loss through NF‐κB activation, impairing muscle function [31]. Experimental evidence further suggests that TNF‐α can impair muscle function by modifying muscle proteins without inducing protein loss [32]. These combined findings highlight the detrimental effects of TNF‐α on muscle performance.

Data on myostatin, a muscle growth suppressor and a member of the myokine family [33], regarding its role in age‐related muscle atrophy in various human cohorts are conflicting. Although studies have reported that higher circulating myostatin levels are associated with better muscle function or physical performance, particularly in men [30, 34], others have shown decreasing circulating myostatin levels with ageing [35]. In our study, myostatin levels within plasma EVs decreased as participants' walking speed increased, reflecting improved muscle performance. Furthermore, in the subgroup analysis restricted to the male cohort, the results were not statistically significant, likely due to the small number of male participants (*n* = 7). This di*screpan*cy could be attributed to our measurement of myostatin in EVs rather than plasma, as well as the predominance of female participants (88.2%) in our cohort.

P3NP, a byproduct of Type 3 collagen synthesis, is more significantly associated with physical performance rather than muscle function and is commonly used as a biomarker for measuring muscle remodelling [36]. We observed that P3NP levels within EVs were downregulated in relation to SPPB scores. This finding is consistent with previous research using the Korean Frailty and Aging Cohort Study (KFACS), which reported an inverse association between SPPB scores and P3NP levels [37].

The implications of our findings are significant for the future of sarcopenia diagnosis and management. We could evaluate multiple biomarkers within EVs by utilizing a real‐world study of a human cohort, which allowed us to prevent batch effects and facilitate easier comparison between biomarkers, resulting in more precise conclusions. Identifying reliable biomarkers within EVs could revolutionize the approach to sarcopenia diagnosis and treatment, providing noninvasive, accessible and cost‐effective methods for early detection and monitoring. This could lay a foundation for more personalized treatment and improved outcomes for patients.

### 4.1 Limitations

This study has several limitations. During the characterization of plasma‐derived EVs, we also evaluated potential contamination from plasma‐abundant proteins, including albumin and IgG. Interestingly, our findings suggest that these proteins may not be external contaminants but intrinsic components of EVs, consistent with previous report [19]. To further elucidate their structural association with EVs, complementary techniques such as immunoelectron microscopy are required.

Also, the predominance of female participants in our cohort limits the generalizability of our findings. Although a male‐only subgroup analysis did not yield statistically significant results, other cohorts with higher male representation showed similar trends, such as no correlations between lean body mass and myostatin, adiponectin or TNF‐α [38]. Furthermore, another male cohort demonstrated a negative correlation between P3NP and gait speed, and meta‐analyses have shown a significant negative correlation between TNF‐α and hand grip strength [27, 28]. Differing results across cohorts highlight the influence of cohort characteristics, with our female‐dominant cohort may account for the differences observed in prior studies. Future studies should aim to include a more balanced gender distribution to validate these findings across different populations. Finally, although we observed significant associations with several EV biomarkers, the exact mechanisms by which these EVs‐associated proteins influence muscle function and performance require further investigation. Thus, longitudinal studies are needed to establish causality and explore the potential of these biomarkers in predicting sarcopenia progression and treatment response.

## 5 Conclusion

Using the OsteoSarcopenia cohort, we performed a proteomic analysis of human plasma‐derived EVs to examine the relationships between sarcopenia biomarkers (myostatin, adiponectin, P3NP, CRP and TNF‐α) and sarcopenia‐related factors (muscle mass, muscle function and muscle performance). Our findings revealed that EVs‐derived myostatin, P3NP and TNF‐α were strongly correlated with sarcopenia‐related factors, specifically muscle function and muscle performance, highlighting the potential of circulating EVs as reliable biomarkers for sarcopenia. By applying biomarker‐specific cutoff values, we identified significant group differences in sarcopenia parameters, further supporting the diagnostic relevance of EVs‐derived biomarkers. These findings highlight the utility of EV biomarkers in identifying and monitoring muscle function and performance impairments in sarcopenia. Such blood‐based diagnostic tests could enable earlier detection and personalized management of sarcopenia, offering a noninvasive and accessible approach for clinical assessment and targeted intervention strategies.

## Conflicts of Interest

The authors declare no conflicts of interest.

## Supporting information


**Table S1** Differences in biomarker levels between high and low muscle function and performance groups.

**Figure S1** Isolation of EVs from human plasma from Osteoporosis Sarcopenia (OsteoSarc) cohort (A) Illustration of the EV isolation method. (B) Western blot images of EV markers and lipoproteins in fractions collected via DGUC from human plasma. The same volume of each fraction was loaded. Ponceau S staining of the membrane after transferring. (C) Western blot images of CD9, Albumin, and IgG in EV sampled collected via DGUC from human plasma, with (−) and without Albumin/IgG depletion. (D) Representative TEM images of plasma‐derived EVs. Scale bars: 50 nm. (E) Particle size of each DGUC fraction (*n* = 3). (F) Particle and protein concentrations of each DGUC fraction (*n* = 3). Data are expressed as mean ± standard error of the mean. DGUC, differential gradient ultracentrifugation; EV, extracellular vesicle; TEM, transmission electron microscopy.
