# Supplementary material for: Plasma Extracellular Vesicles Biomarkers Linked to Lower Muscle Mass, Function and Physical Performance in Sarcopenia
Source: J Cachexia Sarcopenia Muscle. 2025 Mar 31;16(2):e13784. doi: 10.1002/jcsm.13784 (PMC11955922; doi:10.1002/jcsm.13784)
Supplement: Supplementary file 1 — Table S1 Differences in biomarker levels between high and low muscle function and performance groups. [file JCSM-16-e13784-s002.docx]

**Supplementary table** **1. Differences in biomarker levels between high and low muscle function and performance groups**

| **Sarcopenia-related factors** | | **Myostatin (pg/mL)** | | **P3NP (ng/mL)** | | **Adiponectin (ng/mL)** | | **CRP (ug/mL)** | | **TNF-α (pg/mL)** | |
| --- | --- | --- | --- | --- | --- | --- | --- | --- | --- | --- | --- |
|  |  | **Mean ± SD (n)** | ***p-*value** | **Mean ± SD (n)** | ***p-*value** | **Mean ± SD (n)** | ***p-*value** | **Mean ± SD (n)** | ***p-*value** | **Mean ± SD (n)** | ***p-*value** |
| **SARC-F, score** | **High** | **35.6 ± 47.7 (15)** | **0.040** | 0.5 ± 0.2 (17) | 0.574 | 314.2 ± 91.4 (17) | 0.538 | **2.1 ± 0.1 (17)** | **0.004** | 0.1 ± 0.1 (15) | 0.308 |
|  | **Low** | **16.7 ± 12.2 (67)** |  | 0.4 ± 0.0 (76) |  | 304.5 ± 96.9 (76) |  | **1.3 ± 0.1 (76)** |  | 0.1 ± 0.1 (57) |  |
| **Hand grip strength, kg** | **High** | 15.8 ± 11.1 (30) | 0.260 | 0.4 ± 0.0 (39) | 0.549 | 296.5 ± 109.4 (39) | 0.901 | 1.4 ± 0.1 (39) | 0.761 | 0.1 ± 0.0 (30) | 0.824 |
|  | **Low** | 22.7 ± 28.5 (52) |  | 0.5 ± 0.1 (54) |  | 313.2 ± 84.5 (54) |  | 1.4 ± 0.1 (54) |  | 0.1 ± 0.1 (42) |  |
| **Walking speed, m/s** | **High** | 14.6 ± 10.6 (27) | 0.095 | 0.4 ± 0.0 (39) | 1 | 304.9 ± 80.8 (39) | 0.755 | 1.6 ± 0.1 (39) | 0.327 | 0.1 ± 0.0 (25) | 0.731 |
|  | **Low** | 23.4 ± 28.7 (51) |  | 0.5 ± 0.1 (54) |  | 302.6 ± 104.5 (54) |  | 1.3 ± 0.1 (54) |  | 0.1 ± 0.1 (43) |  |
| **SPPB, score** | **High** | 16.8 ± 11.5 (29) | 0.528 | 0.4 ± 0.0 (37) | 0.829 | 285.8 ± 102.2 (37) | 0.157 | 1.4 ± 0.1 (37) | 0.973 | 0.1 ± 0.1 (26) | 0.609 |
|  | **Low** | 22.5 ± 29.3 (49) |  | 0.5 ± 0.1 (52) |  | 316.0 ± 89.6 (52) |  | 1.5 ± 0.1 (52) |  | 0.1 ± 0.1 (42) |  |
| **Five-time-sit-to-stand test, sec** | **High** | 16.2 ± 10.5 (19) | 0.557 | 0.4 ± 0.0 (23) | 0.643 | 299.1 ± 111.8 (23) | 0.933 | 1.6 ± 0.1 (23) | 0.673 | 0.1 ± 0.0 (18) | 0.428 |
|  | **Low** | 21.7 ± 27.2 (59) |  | 0.4 ± 0.1 (66) |  | 305.0 ± 90.2 (66) |  | 1.4 ± 0.1 (66) |  | 0.1 ± 0.1 (50) |  |

SPPB, short physical performance battery; P3NP, procollagen type 3 N-terminal peptide; CRP, C-reactive protein; TNF-α, tumor necrosis factor-alpha. Samples outside the ELISA range in myostatin and TNF-α were excluded. *p*-values were calculated using the Mann-Whitney test.
