# Supplementary material for: Plasma Extracellular Vesicles Biomarkers Linked to Lower Muscle Mass, Function and Physical Performance in Sarcopenia
Source: J Cachexia Sarcopenia Muscle. 2025 Mar 31;16(2):e13784. doi: 10.1002/jcsm.13784 (PMC11955922; doi:10.1002/jcsm.13784)
Supplement: Supplementary file 2 — Figure S1 Isolation of EVs from human plasma from Osteoporosis Sarcopenia (OsteoSarc) cohort (A) Illustration of the EV isolation method. (B) Western blot images of EV markers and lipoproteins in fractions collected via DGUC from human plasma. The same volume of each fraction was loaded. Ponceau S staining of the membrane after transferring. (C) Western blot images of CD9, Albumin, and IgG in EV sampled collected via DGUC from human plasma, with (−) and without Albumin/IgG depletion. (D) Representative TEM images of plasma‐derived EVs. Scale bars: 50 nm. (E) Particle size of each DGUC fraction (n = 3). (F) Particle and protein concentrations of each DGUC fraction (n = 3). Data are expressed as mean ± standard error of the mean. DGUC, differential gradient ultracentrifugation; EV, extracellular vesicle; TEM, transmission electron microscopy. [file JCSM-16-e13784-s001.pdf]

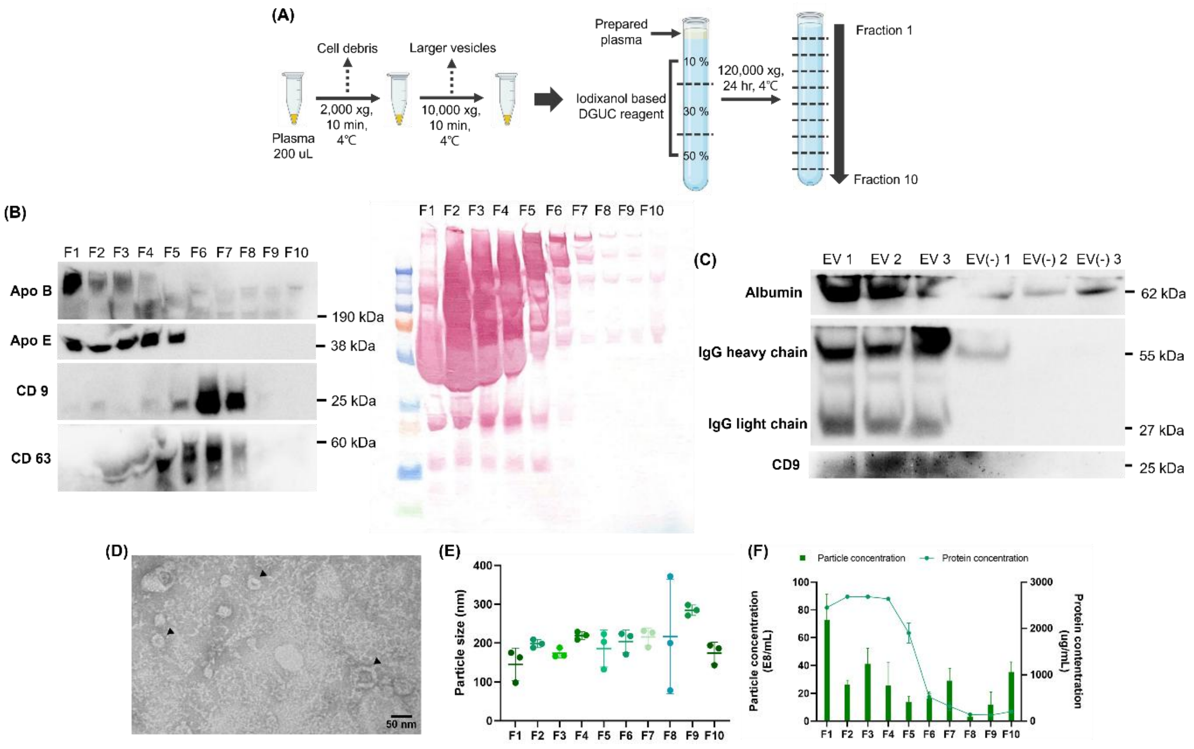

Figure S1. Isolation of EVs from human plasma from Osteoporosis Sarcopenia (OsteoSarc) cohort (A) Illustration of the EV isolation method. (B) Western blot images of EV markers and lipoproteins in fractions collected via DGUC from human plasma. The same volume of each fraction was loaded. Ponceau S staining of the membrane after transferring. (C) Western blot images of CD9, Albumin, and IgG in EV sampled collected via DGUC from human plasma, with (-) and without Albumin/IgG depletion. (D) Representative TEM images of plasma-derived EVs. Scale bars: 50 nm. (E) Particle size of each DGUC fraction (n = 3). (F) Particle and protein concentrations of each DGUC fraction (n = 3). Data are expressed as mean ± standard error of the mean. DGUC, differential gradient ultracentrifugation; EV, extracellular vesicle; TEM, transmission electron microscopy
